# Assessment of Hyperglycemia, Hypoglycemia and Inter-Day Glucose Variability Using Continuous Glucose Monitoring among Diabetic Patients on Chronic Hemodialysis

**DOI:** 10.3390/jcm10184116

**Published:** 2021-09-12

**Authors:** Maria Divani, Panagiotis I. Georgianos, Triantafyllos Didangelos, Vassilios Liakopoulos, Kali Makedou, Fotios Iliadis, Christos Savopoulos, Dimitrios M. Grekas

**Affiliations:** 1 1st Propedeutic Department of Medicine, AHEPA Hospital, Aristotle University of Thessaloniki, 54636 Thessaloniki, Greece; mariadivani@yahoo.com (M.D.); didang@med.auth.gr (T.D.); pangeorgi@gmail.com (F.I.); chrisavopoulos@gmail.com (C.S.); mtnippokratis@yahoo.com (D.M.G.); 2 Hemodialysis Unit, 1st Department of Medicine, AHEPA Hospital, Aristotle University of Thessaloniki, 54626 Thessaloniki, Greece; liakopul@otenet.gr; 3 Laboratory of Biochemistry, AHEPA Hospital, Aristotle University of Thessaloniki, 54636 Thessaloniki, Greece; kalimakedou@gmail.com

**Keywords:** continuous glucose monitoring, glycated albumin, hemodialysis, hemoglobin A1c, time in ranges

## Abstract

Continuous glucose monitoring (CGM) facilitates the assessment of short-term glucose variability and identification of acute excursions of hyper- and hypo-glycemia. Among 37 diabetic hemodialysis patients who underwent 7-day CGM with the iPRO2 device (Medtronic Diabetes, Northridge, CA, USA), we explored the accuracy of glycated albumin (GA) and hemoglobin A1c (HbA1c) in assessing glycemic control, using CGM-derived metrics as the reference standard. In receiver operating characteristic (ROC) analysis, the area under the curve (AUC) in diagnosing a time in the target glucose range of 70–180 mg/dL (TIR^70–180^) in <50% of readings was higher for GA (AUC: 0.878; 95% confidence interval (CI): 0.728–0.962) as compared to HbA1c (AUC: 0.682; 95% CI: 0.508–0.825) (*p* < 0.01). The accuracy of GA (AUC: 0.939; 95% CI: 0.808–0.991) in detecting a time above the target glucose range > 250 mg/dL (TAR^>250^) in >10% of readings did not differ from that of HbA1c (AUC: 0.854; 95% CI: 0.699–0.948) (*p* = 0.16). GA (AUC: 0.712; 95% CI: 0.539–0.848) and HbA1c (AUC: 0.740; 95% CI: 0.570–0.870) had a similarly lower efficiency in detecting a time below target glucose range < 70 mg/dL (TBR^<70^) in >1% of readings (*p* = 0.71). Although the mean glucose levels were similar, the coefficient of variation of glucose recordings (39.2 ± 17.3% vs. 32.0 ± 7.8%, *p* < 0.001) and TBR^<70^ (median (range): 5.6% (0, 25.8) vs. 2.8% (0, 17.9)) were higher during the dialysis-on than during the dialysis-off day. In conclusion, the present study shows that among diabetic hemodialysis patients, GA had higher accuracy than HbA1c in detecting a 7-day CGM-derived TIR^70–180^ < 50%. However, both biomarkers provided an imprecise reflection of acute excursions of hypoglycemia and inter-day glucose variability.

## 1. Introduction

The 2020 Kidney Disease Improving Global Outcomes (KDIGO) guideline recommends the use of hemoglobin A1c (HbA1c) for long-term monitoring of glycemic control among patients with diabetes and chronic kidney disease (CKD) [1]. The periodic measurement of HbA1c remains the standard-of-care despite the fact that the diagnostic accuracy of this glycemic biomarker declines in parallel with the progression of CKD [2]. Notably, the reliability of HbA1c is even lower among end-stage-kidney-disease (ESKD) patients on hemodialysis [3]. In these patients, certain conditions and pathophysiological processes, such as the formation of carbamylated hemoglobin within the context of uremia and metabolic acidosis, the presence of anemia and the use of erythropoietin-stimulating agents or intravenous iron supplements, have long been recognized as factors that limit the accuracy and precision of HbA1c [4]. Alternative glycemic biomarkers, such as glycated albumin (GA), have been proposed, but their adoption for use in daily clinical practice remains low, mainly due to the lack of solid evidence to demonstrate their superiority over HbA1c in assessing glycemic control and in prognosticating the long-term risk for diabetes-related complications and adverse clinical outcomes [5].

Taking into consideration the drawbacks of currently established glycemic biomarkers, the 2020 KDIGO guideline recommends that the use of continuous glucose monitoring (CGM) may offer advantages in self-management of diabetes in individuals with advanced CKD [1], mainly by facilitating the identification of acute excursions of hyper- and hypo-glycemia. Unlike HbA1c and GA, CGM enables the direct observation of short-term (intra- and inter-day) glucose variability [6,7], providing the opportunity for the immediate adjustment of anti-diabetic therapy and/or lifestyle modifications. In 2019, the Advanced Technologies & Treatments for Diabetes (ATTD) [8] released an updated consensus statement aiming to refine core metrics for the assessment of glycemic control that includes three key CGM-derived measurements: (i) the percentage of readings and time per day within target glucose range of 70–180 mg/dL (TIR^70–180^); (ii) the percentage of readings and time above the target glucose range of 250 mg/dL (TAR^>250^); (iii) the percentage of readings and time below the target glucose range of 70 mg/dL (TBR^<70^) [8]. In a combined analysis of four randomized trials incorporating CGM data from 545 adults with type 1 diabetes, it was shown that an HbA1c of 7% and 8% corresponded to a TIR^70–180^ of 70% and 50%, respectively [9]. An average treatment-induced reduction of 0.6% in HbA1c over 6 months was associated with a rise of 10% in TIR^70–180^ [9]. However, the association of HbA1c and GA with CGM-derived metrics of glycemic control among diabetic hemodialysis patients has not been previously investigated.

Therefore, the primary aim of the present study was to explore the accuracy of GA versus HbA1c in assessing hyperglycemia and hypoglycemia among 37 diabetic hemodialysis patients using CGM-derived parameters as reference-standard metrics of glycemic control. A secondary objective was to describe the short-term glucose variability and identify potential differences in glycemic profiles of these patients between the dialysis-on and dialysis-off days.

## 2. Materials and Methods

### 2.1. Study Population

The association of HbA1c and GA with mean 7-day CGM-derived glucose in this cohort has been previously reported elsewhere [10]. The overall study population consisted of 37 diabetic ESKD patients who were receiving renal replacement therapy in 2 dialysis centers in Thessaloniki, Greece (Hemodialysis Unit, AHEPA University Hospital and Tatiana Dialysis Center). Patients were eligible in this study if (i) they had ESKD treated with hemodialysis 3 times weekly for at least 3 months; (ii) they had history of type 1 or type 2 diabetes under treatment with insulin analogs or oral anti-diabetic medications; (iii) their anti-diabetic regimen remained unmodified for at least 3 months prior to study enrollment. The prespecified exclusion criteria of the study were as follows: (i) concurrent systemic inflammatory disease or acute infection during the study assessments; (ii) hospitalization for diabetic ketoacidosis or other acute deregulation of diabetes over the last 3 months; (iii) severe anemia (Hb < 9 g/dL) requiring blood transfusion over the past 3 months; (iv) hypoalbuminemia, defined as predialysis serum albumin <3.5 g/dL; (v) hospitalization for acute coronary syndromes or stroke during the previous 3 months; (vi) history of malignancy or any other medical condition associated with short life expectancy; (viii) inability to understand the protocol and provide written informed consent.

The protocol procedures of our study were accordant with the Declaration of Helsinki and its latest amendments, and all patients provided written informed consent prior to study enrollment. The protocol of our study received approval by the ethics committee of the School of Medicine, Aristotle University of Thessaloniki (code of approval: 298/23.3.2016).

### 2.2. Study Protocol

Eligible patients were instructed to visit their dialysis unit 1 h before their regular mid-week dialysis treatment. Study investigators recorded information about the demographic characteristics; type and duration of diabetes; the primary cause of ESKD; presence of comorbidities; other dialysis-related parameters; and the prescribed medications for the management of diabetes, ESKD and other comorbidities. Anthropometric characteristics were recorded by measuring body weight and height, and body mass index (BMI) was calculated as weight divided by height squared. Blood samples were obtained for the determination of routine predialysis hematological and biochemical parameters as well as the assessment of HbA1c and GA. Subsequently, the glucose biosensor of the iPRO device was inserted into the abdominal wall and the CGM was initiated, as described in detail below [11]. The CGM system was removed 1 h prior to the mid-week dialysis session of the next week. During this period, patients were advised to maintain their usual activities and keep their dietary habits stable. Any changes in the prescribed anti-diabetic regimen or in other dialysis-related parameters were not allowed over the 7-day-long CGM.

### 2.3. Study Evaluations

#### 2.3.1. Continuous Glucose Monitoring

The assessment of hyperglycemia, hypoglycemia and glucose variability was performed for 7 days with the Medtronic iPRO CGM system (Medtronic Diabetes, Northridge, CA, USA). A reverse-type (professional) sensor, blind to the patient and healthcare practitioner in real time, was placed on the abdominal wall or the deltoid area and measured interstitial glucose every 5 min, providing approximately 288 recordings daily with the use of a glucose oxidase method. Neither patients nor physicians had access to the CGM data. At the end of the recording, data from the iPRO2 were transferred to a PC and analyzed by special software. CGM data were extracted as an Excel file and in an Ambulatory Glucose Profile format [11]. The CGM biosensor was calibrated retrospectively using capillary glucose measurements that were obtained by the patients themselves using the One Touch Ultra Easy blood glucose monitor (Johnson & Johnson, New Brunswick, NJ, USA). In detail, patients were asked to measure capillary glucose 4 times daily, and the data from the memory of the glucose monitor were used to calibrate the CGM system. Patients were instructed in the use of this monitor and were asked not to share this device with others. To ensure the validity of the calibration, we used only blood glucose recordings that were stored in the memory of the monitor. Furthermore, the device had no alarm system to notify the patients for acute fluctuations in glucose levels aiming to eliminate any potential influence of CGM data on their dietary habits or intensity of insulin treatment.

Based on the recommendations for the assessment of glycemic control in older/high-risk individuals with type 1 or type 2 diabetes that were provided by the 2019 ATTD consensus statement [8], inadequate glycemic control in the present study was defined as 7-day CGM-derived TIR^70–180^, TAR^>250^, or TBR^<70^ in <50%, >10% and >1% of readings, respectively. These metrics were calculated by analyzing the overall number of interstitial glucose measurements obtained during the 7-day CGM. To evaluate the short-term variation in glycemic profiles, the same CGM-derived metrics were calculated separately for the 24 h periods of the dialysis-on and dialysis-off days.

#### 2.3.2. Measurement of Glycemic Biomarkers

HbA1c was determined with the use of a routine HPLC immunoassay. GA was measured by a direct non-radiolabel enzyme-linked immunosorbent assay (DRG International Inc., Springfield, NJ, USA) [10,12,13]. In this assessment, GA in human plasma bound to an immobilized monoclonal antibody that specifically recognized the glycated moieties on human albumin. After incubation for a fixed time, an enzyme-conjugated polyclonal antibody directed against human albumin was added. After the termination of this reaction, the intensity of the color was read in an ELISA reader at 450 nm [10,12,13]. The GA value was calculated as the percentage of GA relative to total albumin, which was measured with a new bromocresol purple method using the same serum sample [10,12,13].

### 2.4. Statistical Analysis

Continuous variables are expressed as mean ± standard deviation (mean ± SD) or median (range), according to normality of the distribution. Categorical variables are expressed as absolute frequencies and percentages. For the description of baseline characteristics, the study population was divided into 2 groups, according to the level of 7-day TIR^70–180^. For the comparison of continuous variables between the 2 study groups, we used the independent Student’s t test or the Mann–Whitney U-test, according to the normality of distribution of each variable. The chi-squared (χ^2^) test was applied to provide between-group comparisons for categorical variables. For comparison of CGM-derived parameters between the dialysis-on and dialysis-off days, we used the paired Student’s t test or Wilcoxon’s signed rank test, where appropriate. The sensitivity and specificity of HbA1c and GA in detecting a 7-day TIR^70–180^ <50%, a TAR^>250^ >10% and a TBR^<70^ >1% were evaluated using the non-parametric receiver operating characteristic (ROC) curves. The area under the ROC curve (AUC) for each glycemic biomarker was presented with 95% confidence interval (CI). Comparisons of ROC curves were performed to explore the significance of the difference in AUCs between GA and HbA1c. Optimal thresholds for diagnosing inadequate glycemic control were estimated using the Youden index. The Youden index represents a statistical metric of the overall diagnostic efficiency of a technique and was calculated as sensitivity + specificity—1 [14]. The higher the Youden index, the higher the diagnostic accuracy at the cut-off point. Probability values of *p* < 0.05 (two-tailed) were considered statistically significant for all comparisons. Statistical analysis was performed using the Statistical Package for Social Sciences version 23.0 (SPSS Inc., Chicago, IL, USA) and MedCalc, version 20.0 (www.medcalc.org, accessed on 15 June 2021).

## 3. Results

### 3.1. Baseline Characteristics of Study Participants

As shown in Table 1, the present study included 37 diabetic hemodialysis patients (20 males and 17 females) with a mean age of 62.0 ± 17.3 years and a median dialysis vintage of 27 months (range: 3, 160). As expected, the burden of cardiovascular comorbidities in our cohort was high, as all these patients had concomitantly hypertension, 70.3% of them had dyslipidemia, 62.2% had history of coronary artery disease, and 45.9% had history of congestive heart failure. For the management of diabetes, the overall study population was on treatment with insulin analogs. As compared with patients who had a 7-day TIR^70–180^ ≥ 50%, those with a TIR^70–180^ in <50% of readings had higher mean 7-day CGM-derived glucose levels (193.3 ± 47.1 vs. 148.9 ± 24.4 mg/dL, *p* < 0.001), higher TAR^>250^ (median (range): 21.3% (0, 52.1) vs. 4.0% (0, 27.6), *p* < 0.001) and lower TIR^70–180^ (39.7 ± 10.0% vs. 74.9 ± 15.5%, *p* < 0.001). In contrast, the 7-day TBR^<70^ and the coefficient of variation (CV) of glucose readings were similar in both groups. The levels of GA were higher in patients with a TIR^70–180^ < 50% than in those with a TIR^70–180^ in ≥50% of readings (21.9 ± 4.6% vs. 15.0 ± 4.1%, *p* < 0.001). However, the levels of HbA1c did not differ between these two groups (7.1 ± 1.3% vs. 6.3 ± 1.4%, *p* = 0.10).

### 3.2. Accuracy of GA and HbA1c in Assessing Hyper- and Hypo-Glycemia

**Figure 1:**
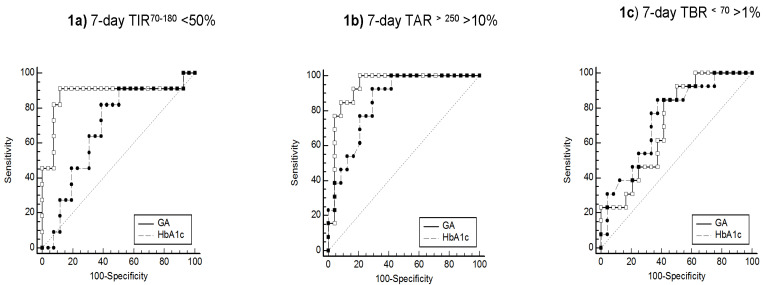
ROC curves of GA and HbA1c in diagnosing inadequate glycemic control using 7-day CGM-derived metrics as the reference standard.

The sensitivity and specificity of GA and HbA1c in assessing hyperglycemia and hypoglycemia are presented in Figure 1 and Table 2. The AUC for GA and HbA1c in diagnosing a 7-day TIR^70–180^ <50% was 0.878 (95% CI: 0.728–0.962) and 0.682 (95% CI: 0.508–0.825), respectively. The AUC for GA was significantly higher than the AUC for HbA1c (difference between areas: 0.196; 95% CI: 0.062–0.330, *p* < 0.01). The optimal diagnostic threshold for GA was >18.96%, which provided 90.9% sensitivity and 88.4% specificity with a Youden index of 0.793. The optimal cut-off point for HbA1c was >6.29%, which did not provide a satisfactory combination of sensitivity (81.8%) and specificity (61.5%) in the detection of a 7-day TIR^70–180^ >50%. Furthermore, the Youden index was 0.433, indicating a lower diagnostic efficiency of HbA1c.

As shown in Figure 1b, the AUC for GA and HbA1c in detecting a 7-day TAR^>250^ > 10% was 0.939 (95% CI: 0.808–0.991) and 0.854 (95% CI: 0.699–0.945), respectively. The difference in AUCs was not statistically significant (difference between areas: 0.085; 95% CI: −0.034 to 0.204, *p* = 0.16). The optimal diagnostic threshold for GA was >16.27% with a Youden index of 0.791, which provided 100% sensitivity and 79.2% specificity. The optimal cut-off point for HbA1c was again >6.29%, providing 92.3% sensitivity and 70.8% specificity with a Youden index of 0.631.

Unlike the high accuracy of these two biomarkers in assessing hyperglycemia, the AUC in detecting a 7-day TBR^<70^ > 1% was 0.712 (95% CI: 0.539–0.848) for GA and 0.740 (95% CI: 0.570–0.870) for HbA1c (Figure 1c). Neither GA nor HbA1c had a satisfactory combination of sensitivity and specificity in detecting a TBR^<70^ in >1% of glucose readings over the 7-day CGM. The Youden index was 0.429 and 0.471 for GA and HbA1c, indicating a similarly lower efficiency of both biomarkers in detecting acute excursions of hypoglycemia (Table 2).

### 3.3. Glucose Variability during the Dialysis-On and Dialysis-Off Days

**Figure 2:**
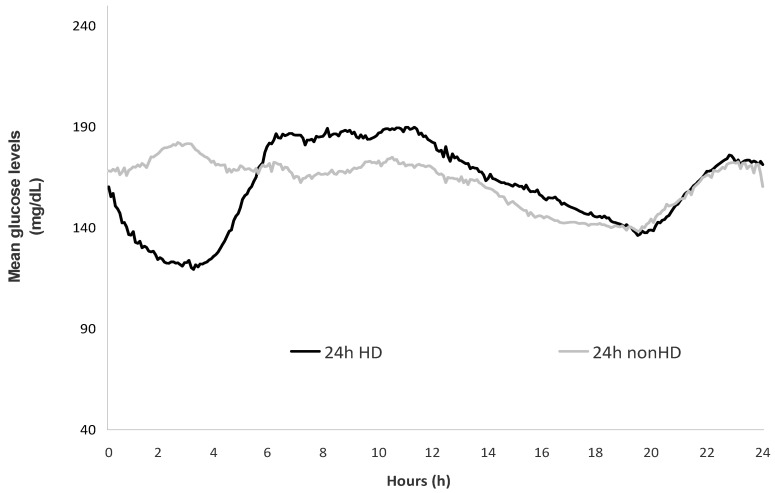
Glucose variation over the 24 h periods of the dialysis-on and dialysis-off days. **Abbreviations:** 24 h HD = dialysis-on day; 24 h nonHD = dialysis-off day.

The glycemic profiles and CGM-derived metrics during the dialysis-on and dialysis-off days are presented in Figure 2 and Table 3. There was no difference between the dialysis-on and dialysis-off days in mean 24 h levels of GGM-derived glucose and percentage of glucose recordings belonging to the ranges of 70–180 and >250 mg/dL. However, 24 h CV of glucose readings (39.2 ± 17.3% vs. 32.0 ± 7.8%, *p* < 0.001) and 24 h CGM-derived TBR^<70^ (median (range): 5.6% (0, 25.8) vs. 2.8 (0, 17.9)) were significantly higher during the dialysis-on than during the dialysis-off days.

## 4. Discussion

The low reliability of HbA1c poses difficulties in the assessment of glycemic control among diabetic hemodialysis patients [4,7]. The measurement of GA is proposed as an alternative glycemic biomarker in this high-risk population [15,16]. In a prior analysis, we showed that GA provided higher accuracy than that of HbA1c in diagnosing a mean 7-day CGM-derived glucose of ≥184 mg/dL [10]. The present study expands our prior observations using three novel CGM-derived parameters as reference-standard metrics of hyperglycemia and hypoglycemia. The main findings of our study are as follows: (i) GA was more accurate than HbA1c in detecting a 7-day TIR^70–180^ in <50% of readings; (ii) in contrast, GA was as accurate as HbA1c in detecting a 7-day TAR^>250^ in >10% of readings; (iii) GA and HbA1c had a similarly low efficiency in diagnosing acute excursions of hypoglycemia, defined as 7-day TBR^<70^ in >1% of readings.

The 2020 KDIGO guideline recommends the periodic evaluation of HbA1c for the management of diabetes in patients with CKD [1], mainly because HbA1c has been extensively validated for the prognostication of adverse diabetes-related complications. However, the validity of CGM-derived parameters as outcome measures is also supported by a growing body of evidence. In a post hoc analysis of 1440 patients with type 1 diabetes enrolled in the Diabetes Control and Complications Trial (DCCT) [17], Beck et al. calculated the TIR^70–180^ using blood glucose concentrations from seven finger-stick measurements obtained during 1 day every 3 months. Each 10% lower TIR^70–180^ was associated with 64% higher risk of retinopathy progression and with 40% higher risk of new-onset microalbuminuria [17]. In a subsequent analysis of the DCCT dataset, biochemical hypoglycemia at cut-off points of <70 and <54 mg/dL was associated with 3-fold and 2.7-fold higher incidence of severe symptomatic hypoglycemia over the subsequent 3 months [18]. In a prospective cohort study, 6,225 Chinese adults with type 2 diabetes were stratified into four groups, according to levels of CGM-derived TIR^70–180^ at baseline [19]. Over 6.9 years of follow-up, as compared with the referent group of TIR^70–180^ >85%, the multivariate-adjusted hazard ratio (HR) for all-cause mortality was 1.23, 1.30 and 1.83 in patients with a TIR^70–180^ 71–85%, 51–70% and ≤50%, respectively [19]. Prospective studies are warranted to explore the prognostic significance of CGM-derived metrics of glycemic control among diabetic hemodialysis patients.

A unique advantage of CGM is that this technique facilitates the assessment of short-term glucose variability and identification of asymptomatic hypoglycemia [8,11]. Prior CGM studies suggested the presence of substantial inter-day glucose variability among diabetic hemodialysis patients, showing that the mean 24 h glucose levels were lower, and episodes of asymptomatic hypoglycemia occurred more commonly during the dialysis-on than during the dialysis-off days [7,20,21,22]. However, these studies had some methodological limitations, such as lack of standardized CGM-derived metrics in data reporting, small sample sizes and short duration of CGM [7,20,21,22]. The present study overcomes these limitations, providing comparisons between 111 pairs of dialysis-on and dialysis-off recordings in a larger sample of 37 hemodialysis patients. In our study, daily glycemic profiles varied considerably despite the fact that the average 24 h glucose levels did not differ between the dialysis-on and dialysis-off days. In particular, the 24 h CV of glucose measurements and TBR^<70^ was significantly higher during the dialysis-on than during the dialysis-off days. These differences were more pronounced between the 4 h period covering the hemodialysis procedure and the corresponding period of the dialysis-off day.

Randomized trials showed that among high-risk or older patients with type 1 or type 2 diabetes, CGM-guided insulin therapy was more effective than usual care in improving glycemic control [23,24]. Among diabetic hemodialysis patients, the use of CGM as a tool to guide therapeutic decisions may be even more beneficial. In a multi-center study enrolling 28 diabetic hemodialysis patients [25], CGM-guided intensification of insulin therapy over 3 months was associated with significant reductions in HbA1c and in mean CGM-derived glucose levels; these benefits were not counteracted by excess risk for symptomatic hypoglycemia [25]. In the DIALYDIAB study [26], 15 diabetic hemodialysis patients entered an initial phase of 6 weeks, during which the management of diabetes was based on usual care. This phase was followed by another period of 6 weeks during which the patients underwent CGM at 2-week intervals. Between the baseline and study-end, significant reductions were observed in CGM-derived glucose and in HbA1c [26]. Larger randomized trials are needed to fully elucidate the potential benefits of CGM-guided therapy in the hemodialysis population.

The strength of the present study is found in its careful evaluation of glycemic control in 37 diabetic hemodialysis patients with the measurement of 2 glycemic biomarkers and the concomitant use of CGM. However, our analysis also has some limitations that need to be acknowledged. The study followed a cross-sectional design, and all examinations were performed on a single occasion. Future studies incorporating repeated measurements over time are necessary to investigate longitudinal associations of HbA1c and GA with the change in CGM-derived parameters. Furthermore, although our work provided much longer CGM data than prior studies [20,21,22], we recognize that even longer sampling periods (i.e., 2 weeks) may be required to precisely capture the variability in daily glycemic profiles in hemodialysis patients [27]. Lastly, we acknowledge that the sample size of our study is relatively small. Thus, larger studies are warranted to confirm or refute our observations.

## 5. Conclusions

In conclusion, the present study shows that among diabetic hemodialysis patients, GA was more accurate than HbA1c in diagnosing inadequate glycemic control, defined as 7-day CGM-derived TIR^70–180^ in <50% of readings. GA and HbA1c could primarily detect hyperglycemia but provided limited information about the acute excursions of hypoglycemia and the day-to-day variability of interstitial glucose recordings. Further research is warranted to fully elucidate whether the use of CGM is superior to usual care in improving the management of diabetes among patients on hemodialysis.

## Figures and Tables

**Table 1:** Baseline characteristics of study participants.

Parameter	Overall	7-Day TIR^70–180^ in ≥50% of Readings	7-Day TIR^70–180^ in <50% of Readings	*p* Value
Number of patients	37	26	11	-
Age (years)	62.0 ± 17.3	64.7 ± 16.6	55.6 ± 17.8	0.16
Male gender (*n*, %)	20 (54.1%)	14 (53.8%)	6 (54.5%)	>0.90
BMI (kg/m^2^)	26.8 ± 4.1	26.2 ± 2.7	28.5 ± 6.2	0.11
Dialysis vintage (months)	27 (3, 160)	27 (3, 120)	24 (3, 160)	0.57
History of hypertension (*n*, %)	37 (100%)	26 (100%)	11 (100%)	-
History of dyslipidemia (*n*, %)	26 (70.3%)	17 (65.4%)	9 (81.8%)	0.44
History of CAD (*n*, %)	23 (62.2%)	16 (61.5%)	7 (63.6%)	>0.90
History of CHF (*n*, %)	17 (45.9%)	14 (53.8%)	(3 (27.3%)	0.17
Hemoglobin (g/dL)	10.9 ± 1.2	11.0 ± 1.1	10.7 ± 1.5	0.49
Predialysis urea (mg/dL)	139.6 ± 32.2	146.3 ± 33.1	124.0 ± 24.7	<0.05
Predialysis creatinine (mg/dL)	7.1 ± 2.7	7.4 ± 2.7	6.4 ± 2.6	0.33
Predialysis glucose (mg/dL)	164.2 ± 67.4	149.4 ± 39.8	199.2 ± 102.3	<0.05
Serum albumin (g/dL)	3.9 ± 0.5	3.9 ± 0.5	4.0 ± 0.3	0.61
HbA1c (%)	6.5 ± 1.4	6.3 ± 1.4	7.1 ± 1.3	0.10
Glycated albumin (%)	17.1 ± 5.2	15.0 ± 4.1	21.9 ± 4.6	<0.001
Mean 7-day CGM-derived glucose (mg/dL)	162.1 ± 38.1	148.9 ± 24.4	193.3 ± 47.1	<0.001
7-day CGM-derived CV (%)	29.5 ± 6.5	28.5 ± 6.8	31.9 ± 5.0	0.09
7-day CGM-derived TIR^70–180^ (% of readings)	64.5 ± 21.5	74.9 ± 15.5	39.7 ± 10.0	<0.001
7-day CGM-derived TBR^<70^ (% of readings)	3.6 (0, 65.8)	2.5 (0, 17.4)	6.4 (0, 65.8)	0.33
7-day CGM-derived TAR^>250^ (% of readings)	9.2 (0, 52.1)	4.0 (0, 27.6)	21.3 (0, 52.1)	<0.001
Insulin therapy (*n*, %)	37 (100%)	26 (100%)	11 (100%)	-
Oral anti-diabetic medication use (*n*, %)	0 (0%)	0 (0%)	0 (0%)	-

**Abbreviations:** BMI = body mass index; CAD = coronary artery disease; CGM = continuous glucose monitoring; CHF = congestive heart failure; CV = coefficient of variation; HbA1c = glycated hemoglobin A1c; TAR = time above target glucose range; TBR = time below target glucose range; TIR = time in target glucose range.

**Table 2:** Thresholds for GA and HbA1c in detecting a 7-day CGM-derived TIR^70–180^, TAR^>250^, or TBR^<70^ in <50%, >10% and >1% of readings.

	TIR^70–180^ < 50%				TAR^>250^ > 10%	
Parameter	GA	HbA1c	GA	HbA1c	GA	HbA1c
Area under ROC curve	0.878	0.682	0.939	0.854	0.712	0.74
(95% CI)	(0.728–0.962)	(0.508–0.825)	(0.808–0.991)	(0.699–0.948)	(0.539–0.848)	(0.570–0.870)
Youden index (diagnostic efficiency)	0.793	0.433	0.791	0.631	0.429	0.471
Threshold (%)	>18.96	>6.29	>16.27	>6.29	≤18.5	≤6.3
Sensitivity (%)	90.9	81.8	100	92.3	84.6	84.6

**Abbreviations:** CI = confidence interval; GA = glycated albumin; HbA1c = hemoglobin A1c; ROC = receiver operating characteristic; TAR = time above target glucose range; TBR= time below target glucose range; TIR = time in target glucose range.

**Table 3:** Comparison of CGM-derived parameters between dialysis-on and dialysis-off days.

Parameter	Dialysis-On Day	Dialysis-Off Day	*p* Value
Mean 24 h glucose (mg/dL)	159.2 ± 39.6	162.4 ± 47.0	0.39
24 h CGM-derived CV (%)	39.2 ± 17.3	32.0 ± 7.8	<0.001
24 h CGM-derived TBR^<70^ (% of readings)	5.6 (0, 25.8)	2.8 (0, 17.9)	<0.001
24 h CGM-derived TIR^70–180^ (% of readings)	62.2 ± 22.3	65.2 ± 27.5	0.21
24 h CGM-derived TAR^>250^ (% of readings)	9.1 (0, 52.4)	9.4 (0, 55.5)	0.63

**Abbreviations:** CGM = continuous glucose monitoring; CV = coefficient of variation; TAR = time above range; TBR= time below range; TIR = time in range.

## Data Availability

Data available upon request due to restrictions, e.g., privacy or ethical.
